# Differential Tafel Analysis: A Quick and Robust Tool to Inspect and Benchmark Charge Transfer in Electrocatalysis

**DOI:** 10.1021/acscatal.2c03581

**Published:** 2022-10-27

**Authors:** Manuel Corva, Niclas Blanc, Christoph J. Bondue, Kristina Tschulik

**Affiliations:** † Analytical Chemistry II, Faculty of Chemistry and Biochemistry, Ruhr University Bochum, Bochum44780, Germany; ‡ Max-Planck-Institut für Eisenforschung, Max-Planck-Straße 1, Düsseldorf40237, Germany

## Introduction

For decades, the capability to control and convert electric energy into chemical energy has inspired many technological developments and fundamental research works. In particular, electrocatalysts received great attention as tools for an efficient conversion of raw materials to high-value chemicals and fuels, promoting the imperative global transition toward greener energy cycles. Fuel cells and electrolyzers created a new paradigm, and electrocatalysts have shown new paths for the smart exploitation of low-cost or even waste materials, like N_2_ or CO_2_.^1^ At the core of electrochemical conversions, a redox process occurs at an electrode, that is at the interface between an electron conductor and an ion conducting phase. In many applications, the former is a solid and is submerged into the latter, which is an electrolyte-containing liquid solution. When a suitable potential is applied, charge is transferred and electrochemical reactions occur.^2^ Thus, valuable information about the rate of material conversion can directly be obtained by inspecting the current–potential dependence. However, the measured electrochemical current is the result of the complex interplay of different phenomena and mechanisms, and not all of them relate to the intrinsic catalytic properties of the electrocatalyst of interest.^3,4^ Some of them simply sum to the overall detected current while not contributing to the actual chemical conversion. These are usually referred to as nonfaradaic currents, and capacitive currents are a foremost example.^3,5,6^ Other mechanisms instead directly affect, and usually limit, the electrocatalytic performance. Examples are the formation of gas bubbles at the electrode surface and mass-transport effects.^3,7^ However, both nonfaradaic and current-limiting phenomena are unrelated to the intrinsic catalytic performance of the material under study, as they depend on other (external) processes and can be reduced by appropriate device engineering once a high-performance catalyst material has been identified. To identify such catalysts, the electron transfer at the electrode is of particular interest, as its potential dependence is crucial for the applicability of novel catalytic materials. In fact, it is highly desirable for the reaction of interest to occur at the lowest possible potential and at the highest possible rate (that is current) for an efficient electric-to-chemical energy conversion and to meet the standards of large-scale production.

The description of the electron-transfer rate was developed in the first half of the 20th century through the efforts of Tafel, Butler, Volmer, Erdey-Grùz, and Gurney.^8−10^ These pioneers recognized an exponential dependence of electrochemical currents upon the applied potential and attributed it to the electron-transfer process at the electrode. Moreover, they proposed the total current to depend on the sum of two independent and opposite contributions. This has been summarized in the famous Butler–Volmer (BV) equation, frequently used to describe the electron-transfer rate under the assumption of a constant surface concentration of reactants.^8,11^

While the BV equation appears across different fields in different forms,^8^ all of them describe the kinetic current density *j*_K_ (not limited by mass transport) as the sum of an anodic (*j*_a_) and a cathodic (*j*_c_) contribution. These are modeled as exponential functions of the applied potential *E* through two charge-transfer coefficients α_*i*_, according to
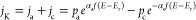
1where the potential of reference *E*_r_ and the parameters *p*_*i*_ depend on the specific formulation of choice (we refer to Dickinson and Wain^8^ for additional details) and  is used for brevity, where *F* and *R* refer to the Faraday and gas constant, respectively, and *T* is the temperature.^8^ Notably, the α_*i*_ coefficients in eq 1 are independent of the potential of reference *E*_r_.^8^ Moreover, IUPAC recommendations highlight how they should be considered unbound by any mechanistic assumptions, i.e., without assuming to know the actual rate-determining step and the number of transferred electrons.^11^ This recommendation results from the fact that, in most cases, the electron-exchange rate at the electrode is the result of multiple reaction steps involving consequent single-electron transfers.^7,11^ Interestingly, these can depend on surface adsorption/diffusion processes and on the reactant/product coverage at the electrode, as considered in microkinetic considerations (i.e., estimations of the theoretical conversion rate corresponding to specific rate-determining steps).^2,5,9,12^ Many of these investigations suggest that the apparent charge-transfer process can be greatly influenced by the specific surface reaction; e.g., the Tafel, Heyrovsky, and Volmer paths for the hydrogen evolution reaction (HER) correspond to specific charge-transfer coefficients.^2,5^ In addition, some works suggest that α_*i*_ might change as a function of potential due to changes in the reactant/product surface coverage, resulting in different rate-determining steps.^2,13^ In this sense, by investigating the potential dependence of α_*i*_ (and of the corresponding Tafel slope) over extended potential ranges, it would be possible to better elucidate the mechanisms determining electrocatalytic activities.^14,15^ However, this would require one to reliably measure α_*i*_ over wide potential ranges, relying on a straightforward and robust approach capable of also determining whenever the exponential model does not hold and more complex processes actually rule the overall reaction rate. Such an approach would likely grant valuable, unambiguous information on the reaction mechanisms occurring at a catalyst during electrocatalytic conversions.

In addition, the exploitation of current derivatives will be an important step toward widely applicable protocols for electrochemical data mining. The mining process relies in fact on the quality of the available data sets and greatly benefits from quick and unbiased screening/clustering methodologies.^16^ While it would be demanding for an algorithm to address, e.g., via fitting, the zero order background correction or the actual exponential character of thousands of current–potential curves, such procedures might be boosted by applying the differentiation methodologies described below.

## Materials and Methods

### Chemicals

Cobalt acetate tetrahydrate (Alfa Aesar, Kandel, Germany, metal basis 99.999%), potassium hydroxide (Alfa Aesar, Kandel, Germany, 85% min., metal basis 99.99%), sodium hydroxide (Sigma-Aldrich, Darmstadt, Germany, 99.99%), and potassium chloride (Sigma-Aldrich, Darmstadt, Germany, 99%) were used without further purification. Pt wire (10 μm diameter, 99.9%) was purchased from ChemPUR (Karlsruhe, Germany).

### Electrochemical Measurements

All electrochemical measurements were performed with a three-electrode system connected to a Bio-Logic VSP 300 electrochemical workstation. A carbon rod electrode was used as counter electrode, and a Ag/AgCl (3 M KCl), Hg/HgO (1.0 M KOH), or Hg/Hg_2_SO_4_ (0.5 M H_2_SO_4_) electrode acted as the reference electrode for measurements performed, respectively, in KCl, KOH, or H_2_SO_4_ containing solutions. For all experiments, a double junction was also used to avoid contamination of and from the reference electrode. Details about the conversion of the potentials to the reversible hydrogen electrode (RHE) can be found in the Supporting Information (SI) in Section SI.1.

### Microelectrode Preparation

Platinum microelectrodes of 10 μm diameter were built by thermally sealing a Pt wire (length ca. 1 cm, 10 μm diameter) into a soda lime glass capillary under vacuum. The Pt wire was then soldered inside the capillary to a silver wire serving as an electrical contact. The tip of the Pt microelectrode was then polished to obtain a clean and smooth surface by polishing with alumina slurries of decreasing grain size (1, 0.3, and 0.05 μm).

### Numerical Simulations

Numerical simulations were performed with the commercially available finite element solver COMSOL Multiphysics 5. The simulations of the voltammetric responses at the microelectrodes were performed in a two-dimensional model geometry with axial symmetry representing a hemispherical space (see details in Figure S1). The electrode is located at the bottom in the center of the quarter circle geometry. As this model represents a microelectrode embedded in an insulating substance, the lower boundary is represented by an inert no-flux boundary.

The outer boundary was set to bulk concentration, and diffusion was assumed as the only mass transport type. The electrochemical reaction was simulated using a Nernst-equation equilibrium potential and classic Butler–Volmer kinetics.

### DEMS Measurements

Differential electrochemical mass spectrometry (DEMS) measurements were done on a home-built setup following the design principle introduced by Wolter and Heitbaum.^17^ In brief, the system features a differentially pumped mass spectrometer (QMA 410, Pfeiffer Vacuum). This allows us to keep the pressure in the ion source (cross beam ion source with magnets, yttriated iridium) at 1 × 10^–4^ mbar, whereas the pressure in the mass filter and the secondary electron multiplier is kept at 2 × 10^–6^ mbar during the measurement. The section of the vacuum system that features the ion source is connected to the electrochemical cell, where a porous PTFE membrane (Pall Inc., PTF002LH0P – SAMP) creates an interface between the liquid electrolyte and the vacuum. Due to a pore size of 20 nm, the hydrophobic properties of PTFE, and the high surface tension of water, aqueous electrolytes cannot penetrate the pores.^17^

The employed electrochemical cell is the dual thin layer cell that was introduced by Jusys et al.^18^ and discussed in depth by Baltruschat.^19^ The cell is a two-compartment flow cell where the electrochemical reaction occurs in the first compartment. The electrolyte then flows to the second compartment where it reaches the vacuum/electrolyte interface. Volatile products of the electrochemical reaction dissolved in the electrolyte evaporate into the vacuum and are detected by the mass spectrometer.

We used standard settings for the ion source: the potential of the formation room was set to 100 V above ground and the potential of the filaments was 70 V lower than that of the formation room (i.e., 30 V above ground).

### Data Treatment and Analysis

Analysis of the collected experimental data and simulation results was performed by OriginPro 2021 (64-bit). The same software has been used to calculate and plot the analytical model described in Section SI.5. Differentiation was performed after applying a 20 point Savitzky–Golay smoothing (2nd order), if not otherwise stated.

## Theoretical Basis

### The Limits of Tafel Analyses

To develop a reliable method to extract the charge-transfer coefficients α_*i*_, we first have to consider that eq 1 does not explicitly describe the possible potential dependence of the parameter terms *p*_*i*_ (where *i* refers to either cathodic *c* or anodic *a* terms), which are routinely assumed to be constant. Due to this, a fitting of experimental current densities by means of eq 1 can lead to erroneous results due to either oversimplification (pre-exponential factors *p*_*i*_ are considered to be constant) or overparametrization (if more complex formulations are assumed). However, it is possible to make a first step toward a reliable estimation of the α_*i*_ values by inspecting the (natural) logarithm of the current. In fact, if a generic quantity (*X*) shows a linear dependence over a specific potential region in its ln(*X*) – *E* plot, that is
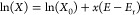
2then *X* can be described by
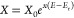
3over the considered potential region. Thus, exponential dependence can be assumed with good approximation, and linear fitting of eq 2 can provide information about *x*. This approach represents the core of the Tafel analysis, an approach based on the empirical work of Tafel,^2,5,20^ and provides a practical path for the determination of α_*i*_ from measured current data. One should note that, if we are only interested in extracting α_*i*_ in eq 1, then current densities *j*_*i*_ or currents *I*_*i*_ (*I*_*i*_ = *Aj*_*i*_) are equivalent under the assumption of a potential-independent electroactive area *A*.

**Figure 1:**
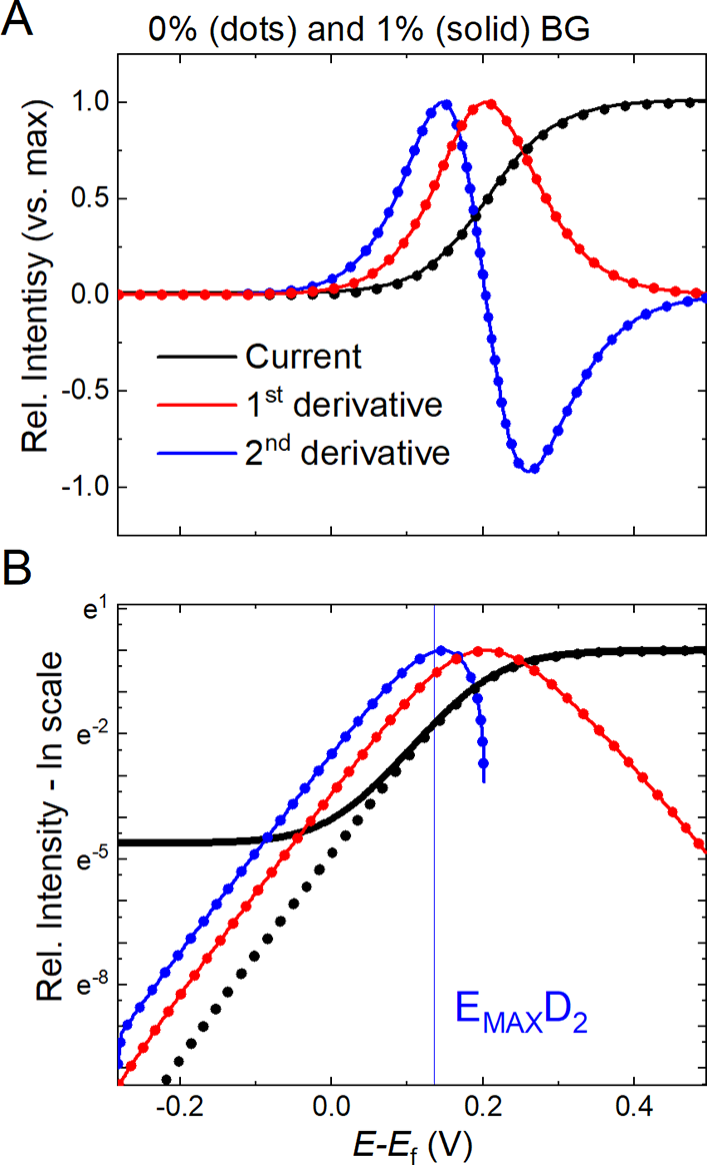
(A) Normalized simulated LSV response of a one-electron oxidation (black) without (dots) and with (solid line) the addition of a constant background (BG). Corresponding first (red) and second (blue) normalized derivatives are included; their maximum highlights the end of the kinetically controlled contribution to the simulated current. (B) Corresponding semilogarithmic plots. The presence of mass-transport limitations and of constant background contributions causes a deviation from the expected linear dependence. Upon derivation, distortions due to mass-transport limitations are clearly revealed and constant background contributions are automatically removed, allowing to identify a more ideal Tafel region.

If only the described electron transfer process contributes to the rate of conversion, then the measured current is fully described by the kinetic contribution, that is *I* = *I*_K_. Then, if one of the two *I*_*i*_ contributions can be neglected, then eq 3 can be used to describe the total current *I*. Additional details are provided in Section SI.2 and in further literature works.^7,21^ If the total current is indeed correctly described by eq 3, then a straight line has to be observed in the ln(*I*) – *E* plot. Unfortunately, only few experimental designs (very low scan rates, small exposed surface, enhanced mass transport) allow the measurement of electrocatalytic currents affected solely by kinetic contributions. Practically, many other contributions, like mass transport limitations and capacitive currents, additionally affect the measured current *I*. To highlight their effects, we report finite element simulations of the current response of a single-electron oxidation occurring at an ultramicroelectrode (UME) in Figure 1. We set *E*_r_ = *E*_f_, where *E*_f_ is the formal potential,^8^ and define the overpotential as η = *E* – *E*_f_. Thus, we refer to higher (smaller) overpotentials only if they are increasing (decreasing) along the positive (negative) direction. We are going to consider in the next section what happens at “high” and “low” overpotentials, where “high” or “low” is determined by the magnitude of the kinetic current with respect to either the limits of mass transport or any nonfaradaic contribution, respectively. Importantly, while it is not easy to define these reference values *a priori*, this will not limit the quality of the extracted information, as we will show below.

First, we consider the current response at sufficiently high overpotentials: the exponential growth is limited by mass transport, the semilogarithmic current plot in Figure 1B deviates from a straight line, and a steady state current is reached. We exploit the maximum current value *I*_max_ to normalize the current data according to
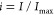
4and move to relative intensities. Normalization to *I*_max_, that is the maximum current value over the investigated range, instead of the steady state value will be useful later in the discussion, when nonideal systems, e.g., not presenting a clear steady state condition, will be considered. Then, we consider the low overpotential limit of Figure 1A. Here, the kinetic contribution can be negligible with respect to other contributions, for instance, capacitive currents. To showcase this, a constant 1% offset has been added to the normalized current *i*: a clear deviation of the offset current (solid line) with respect to the background-free data (dotted line) is observed in the semilogarithmic plot in Figure 1B, even though is it hardly noticeable in Figure 1A and might be overlooked. As distortions emerge both at “high” or “low” overpotential, either mass-transport or nonfaradaic contributions can limit the accessible range for linear fitting and, thus, α_*i*_ estimation by means of a standard Tafel approach. Proper estimates first require the compensation for these contributions.^7,21^ Possible solutions in this sense are the extraction of the kinetic information at “high” overpotentials by manipulation of the Koutechky-Levich equation and adequate background subtractions to extend the limit at “low” overpotentials.^2,6,21^ However, these solutions have some limitations. On the one hand, mass-transport corrections can be applied only under specific conditions, e.g., when the limiting current is known.^21^ On the other hand, constant backgrounds unrelated to the exponential (charge transfer) process must be estimated outside the kinetic range, e.g., before the kinetic contribution offset, and then extended to the potential range under investigation. If other processes alter the evaluation of such backgrounds, such compensations are hardly accessible. A case study is discussed in Section SI.3 for clarification. In addition, the semilogarithmic plots obtained from mass-transport and background corrections are lacking any control parameter to evaluate the quality of the followed procedures. That is, it is possible for the implemented corrections to distort the logarithmic plot to a subtle yet sufficient extent such that wrong charge-transfer coefficients can be derived. To confirm this and affirm the quality of the obtained linear profiles, goodness-of-fit parameters might be inspected and reported. However, they can only certify the quality of the first order polynomial fit, while providing only limited information on the quality of the obtained charge-transfer coefficient α_*i*_.^4^ We refer the reader to Section SI.4, where some potential limits of R^2^-based evaluations are showcased. To cope with these issues, differential Tafel plots (DTPs) have been recently suggested, enabling a more precise and sensitive evaluation of kinetic parameters.^7^ In DTPs, the IUPAC definition of α_*i*_ is exploited: the ln(*j*) – *E* Tafel plot is differentiated, and the value of α_*i*_ is read from the plot. The authors clearly show how it is possible to bypass some debatable aspects of linear fitting and reach a simple and instructive display of α_*i*_ values. However, the method is quite sensitive to incorrect compensations of nonexponential contributions (see Section SI.5 and Figures S5 and S6) and still lacks an external control parameter.

### Exponential Slopes upon Derivation

To tackle these issues, we herein suggest the introduction of the derivative of the current ((∂*i*/∂*E* or ∂*E*_*i*_) as a user-independent, unbiased reference to routinely support the extraction of α_*i*_. The basic concepts of the approach, which will be referred to in the following as differential tafel analysis (DTA), and its comparison to Tafel analysis and DTP are summarized in Table 1.

**Table 1:** Key Concepts of Tafel Analysis, the Differential Tafel Plot (DTP) Method, and Differential Tafel Analysis (DTA)

Tafel/Butler–Volmer assumption	*I*_K_ = *pe*^α*f*(*E* – *E*_r_)^
idea:	the logarithmic plot of an exponential is a first order polynomial, which can be fit to extract the α*f* value
Tafel analysis	ln(*I*_K_) = const + α*fE*
	linear fitting of ln(*I*_K_) vs *E* can provide the value of α*f*
idea:	as ln(*I*_K_) is a first order polynomial in *E*, α*f* is equal to the derivative of ln(*I*_K_)
differential Tafel plot (DTP)	∂_E_ln(*I*_K_) = α*f*
	the value of α*f* can be immediately read in a ∂_*E*_ln(*I*_K_) vs *E* plot
idea:	the derivative of an exponential presents the same exponential profile; the logarithmic plot of both must be a first order polynomial where the slope is identical for both; this must be true if the Tafel/Butler–Volmer assumption holds
derivative of exponential	∂_E_*I*_K_ = *p*α*fe*^α*f*(*E* – *E*_r_)^
differential Tafel analysis (DTA)	ln(*I*_K_) = const + α*fE*
	ln(∂_E_*I*_K_) = const + α*fE*
	linear fitting of ln(*I*_K_) vs *E* and of ∂_E_ln(*I*_K_) vs *E* provides the value of α*f* and, if consistent, proves that the Tafel/Butler–Volmer assumption holds

The concept of the DTA methodology is based on the properties of exponential functions, which preserve their exponential profile upon derivation. Thus, if the measured current can be modeled by eq 3, then its derivatives (*D*_*m*_) can be described by
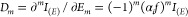
5where *m* refers to the derivative order and the minus sign is required if cathodic contributions are considered. In the following, we will refer to the normalized derivative
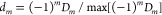
6to more easily compare it with the normalized current *i*. An example is reported in Figure 1A, where the first derivative *d*_1_ is compared with the normalized current *i* to discern the potential region of the fastest current increase. Following the suggested normalizations, it is possible to plot all quantities in the same graph to facilitate comparisons. For increasing overpotential, the derivative initially raises monotonically as expected from an exponential function. This holds up to a maximum value, after which the monotonic increase of the derivative stops. This is due to the increasing mass transport limitations. Intriguingly, the second order derivative *d*_2_ presents a similar behavior with respect to increasing overpotentials, and the end of the monotonic (exponential) increase is even more sensitive to the increasing mass transport limits. Higher derivatives are progressively more sensitive to experimental noise and might therefore not be suitable for our general approach.^22^ We will not delve into further discussion about current derivative profiles (e.g., maxima, zero, and minima corresponding to the inflection points), as they have been already discussed in other works^23^ and are routinely considered during differential pulse and square-wave voltammetry (DPV and SWV, respectively) measurements.^24,25^ Instead, we will target additional information that has not yet been discussed in the literature, to the best of our knowledge.

**Scheme 1:**
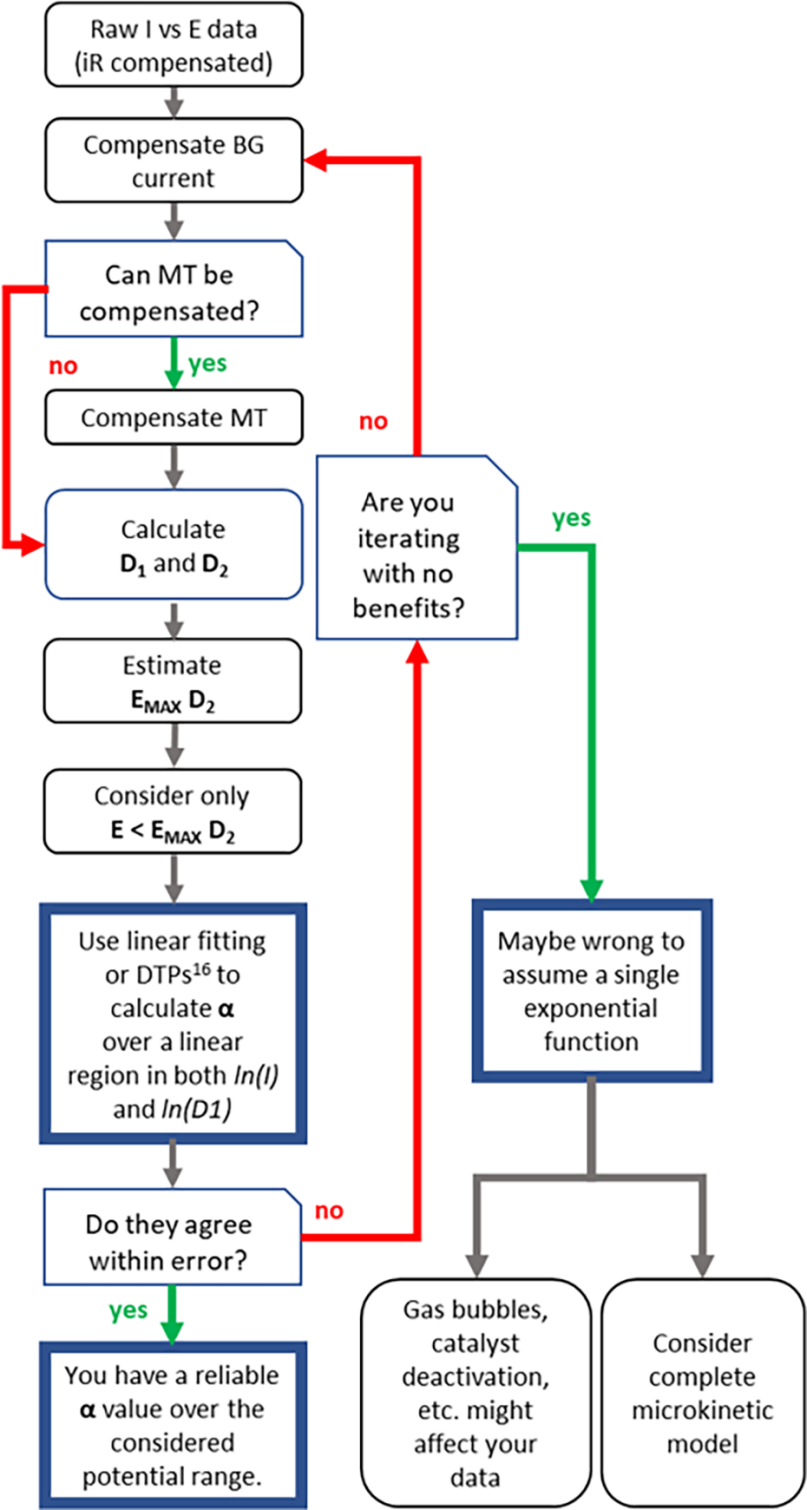
Suggested Workflow for Differential Tafel Analysis To allow easier graphical comparisons, the exchange of *I*, *D*_1_, and *D*_2_ with normalized values by means of eqs 4 and 6 is possible without affecting the workflow. Background (BG) and mass transport (MT) have been abbreviated for clarity.

**Figure 2:**
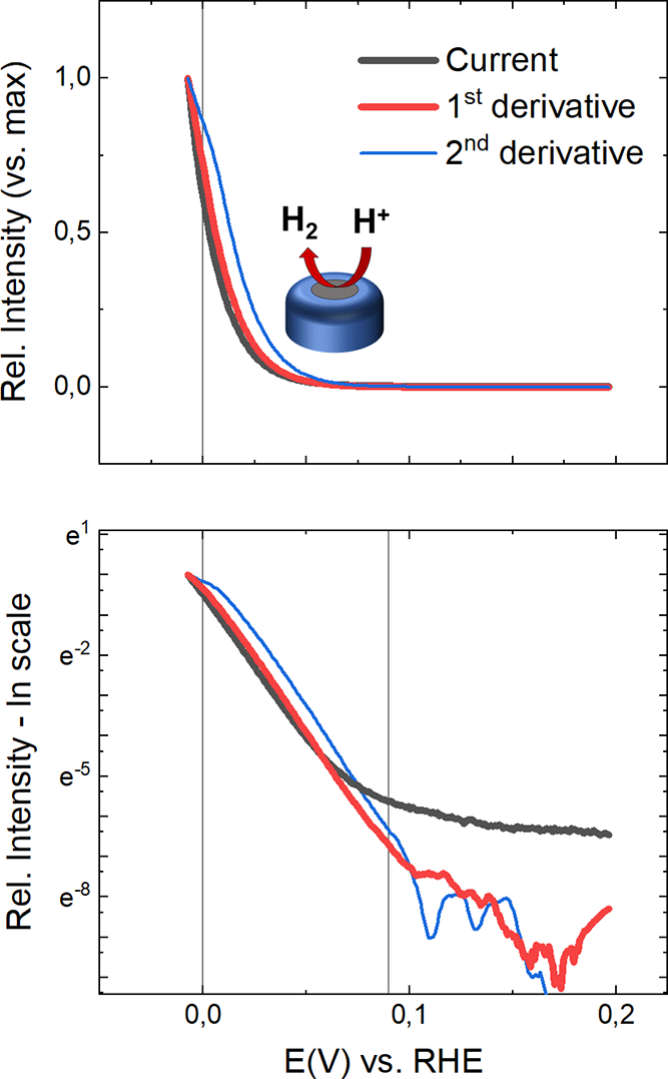
Normalized experimental LSV data (10 mV/s scan rate, 1 min equilibration at OCP) collected from a 10 μm Pt UME in a 500 mM H_2_SO_4_ deaerated solution are reported in black. Normalized first and second derivatives are reported in red and blue, respectively. Corresponding semilogarithmic plots are reported in the lower panel. The potential range considered to extract α*_i_* values is shown by vertical lines. Estimated α_*i*_*f* values obtained from linear fitting of the semilogarithmic plots of current and first derivative data (with the corresponding standard deviation) are reported in Table 1.

If only kinetic contributions rule the total current, then *i*, *d*_1_, and *d*_2_ must have the same slopes in the ln(*X*) – *E* plots. This stems from their identical exponential parameters according to eq 5 (also see Section SI.6). Remarkably, the inverse reasoning holds as well: only exponential functions are preserved upon derivation. So, if ln(*i*) and ln(*d*_*m*_) are parallel, then a clear exponential (kinetic) term must contribute to the total current. We can therefore provide a strong proof that the considered current data are indeed governed by kinetics over a specific potential range. In addition, any constant contribution summing to (and eventually hiding) the kinetic term is automatically canceled upon first derivation, and this extends to linear contributions upon second derivation. Thus, *d*_1_ and *d*_2_ can not only be used as sensible markers to identify the ranges of kinetically controlled regions but also allow straightforward first order background corrections. In contrast to typical Tafel analyses, where it is up to the user to determine suitable potential ranges and appropriate background and/or mass-transport corrections, current derivatives are obtained through well-established algorithms and can be easily compared between different laboratories, acting as a benchmark to prove the validity of the performed analyses and perfectly corroborating DTP approaches. The suggested workflow is indicated in Scheme 1 where the second order derivative (the potential at which it reaches its maximum value, *E*_max_*D*_2_) is suggested as the upper limit for the potential range to use for the linear fitting of ln(*i*) – *E* plots. Depending on the data quality (noise level, presence of discontinuities due to bubble formation, loss of active material, etc.), also the third derivative may be inspected. However, valuable information can already be obtained by analyzing the exponential trend of the first derivative (*D*_1_) while considering the maximum of the second derivative (i.e., its potential *E*_max_*D*_2_) as an upper limit for the available fitting range. Such an upper limit is suggested considering the model case of a single exponential term limited by mass transport, as depicted in Figure 1. In front of more complex systems exhibiting clear slope changes in the ln(*i*) – *D*_2_ plot, this limit should be more carefully considered (as has been done in Figure 2).

## Experimental Results and Discussion

Considering the theoretical framework discussed above, we thus introduce the analysis of current derivatives as an unambiguous tool to corroborate the estimation of charge-transfer coefficients. We showcase this by means of different experimental examples in which we consider linear sweep voltammetry (LSV) measurements targeting the charge-transfer coefficients of the HER and the oxygen evolution reaction (OER) on a 10 μm diameter Pt UME in Ar-saturated solutions. The goal is to demonstrate, not only for analytical solutions or numerical simulations, that the derivative of the current allows one to reliably investigate electrocatalytic currents. We will showcase for three case studies how differentiation can bring important kinetic information and help to model which processes might occur at the electrode.

**Figure 3:**
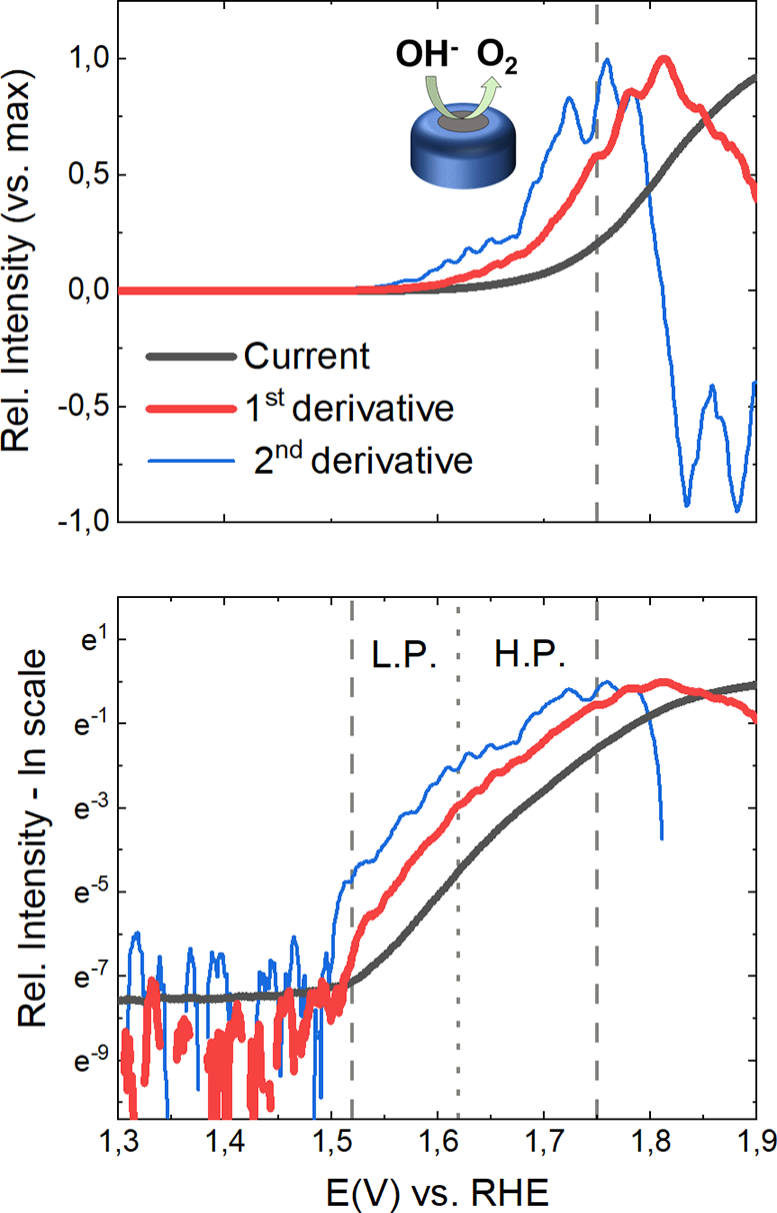
Normalized experimental LSV data (10 mV/s scan rate, 1 min equilibration at OCP) collected from a 10 μm Pt UME in a 1.0 M KOH deaerated solution are reported in black. Normalized first and second derivatives are reported in red and blue, respectively. To evaluate the second derivative, the point window has been increased from 20 to 60 to reduce the effect of noise. Corresponding semilogarithmic plots are reported in the lower panel. The potential ranges considered to extract α_*i*_ values are shown by vertical lines. Estimated α_*i*_*f* values obtained from a linear fitting of the semilogarithmic plots of the current and first derivative data with the corresponding standard deviation are reported in Table 1. Two linear regions are observed and independently fitted.

We first report the electrocatalytic currents collected in 0.5 M H_2_SO_4_ and 1.0 M KOH solutions, respectively, in Figures 2 and 3. As discussed, these currents and their derivatives have been normalized to their maximum value for the ease of comparison (see eq 6). As a first step, the profiles of the first and second derivative (*d*_1_ and *d*_2_, respectively) are inspected for the HER process, highlighting a clear exponential dependence on the applied potential. Straight lines are observed in the semilogarithmic plots, whereas noise fluctuations affect the derivatives at more positive potentials. As no correction has been applied to the collected experimental data, the natural logarithm of the current (ln *i*) shows a strong deviation from a linear dependence at more positive potentials (that is when kinetic contributions become comparable or smaller than the background current).

A different behavior is observed for the OER data (Figure 3), where not only two different slopes are observed at increasing applied potentials but also a clear maximum is visible for both *d*_1_ and *d*_2_. These differences are likely related to the more complex reaction pathway involving multiple reaction steps.^26,27^ Intriguingly, these observations suggest that two different phenomena characterize the electrocatalytic response at increasing potentials, while at around 1.62 V vs RHE, the charge-transfer coefficient decreases to 59% of its initial value and allows us to clearly distinguish a low-potential and a high-potential region (L.P. and H.P. in Figure 3, respectively). A predominant kinetic control is suggested for both regions, as both the current and its derivative show a linear trend and are parallel to each other. This suggests two different exponential trends and matches previous works reporting two different Tafel values for OER at Pt at lower and higher potentials.^26,27^ However, above 1.75 V, the maxima in *d*_1_ and *d*_2_ resemble the case shown in Figure 1 and suggest a clear change: the current no longer monotonically increases, thus suggesting that electron transfer is no longer the predominant contribution. This might originate from severe mass-transport limitations due to the smaller diffusion coefficient of hydroxide ions if compared to protons.^28^ Alternatively, a change in the rate-determining step might limit the electron transfer process itself.^12,27^ The transformation and restructuring of Pt surfaces in alkaline conditions during the OER might be a third reason, where a drastic change of surface activity might follow surface oxidation.^29^ Notably, it is not straightforward to assign the reason for the observed changes in Tafel slopes for OER and what is the actual limiting mechanism above 1.75 V based on the experimental current data only. However, the Tafel slope values extracted from the charge-transfer coefficients obtained from the semilogarithmic plots of the first derivative agree with reported literature values for polycrystalline Pt in acidic and alkaline conditions (see Table 2). This further showcases the effectiveness of considering the current derivatives in kinetic investigations to extract indicative parameters for even more complex reactions (e.g., OER on Pt).

**Table 2:** Exponential Slopes from Linear Fitting of Semi-logarithmic Plots

	α_*i*_*f* (V^–1^)		Tafel slope (mV/dec)^a^		
	*d*_1_	*i*	*d*_1_	*i*	literature
0.5 M H_2_SO_4_	73.4 ± 0.1	60.1 ± 0.8	31	38	30^2,30^
1.0 M KOH, low potential (L.P.)	31.2 ± 0.1	29.1 ± 0.1	74	79	70^5,26,27^
1.0 M KOH, high potential (H.P.)	18.4 ± 0.1	21.0 ± 0.1	125	110	120^5,26,27^

a Exponential slopes extracted from the natural logarithm of the first derivative (*d*_1_) and current (*i*) data of Figures 2 and 3 are converted to the corresponding Tafel slopes (TfS) according to
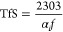
which is a straightforward mathematical derivation.^5,30^

**Figure 4:**
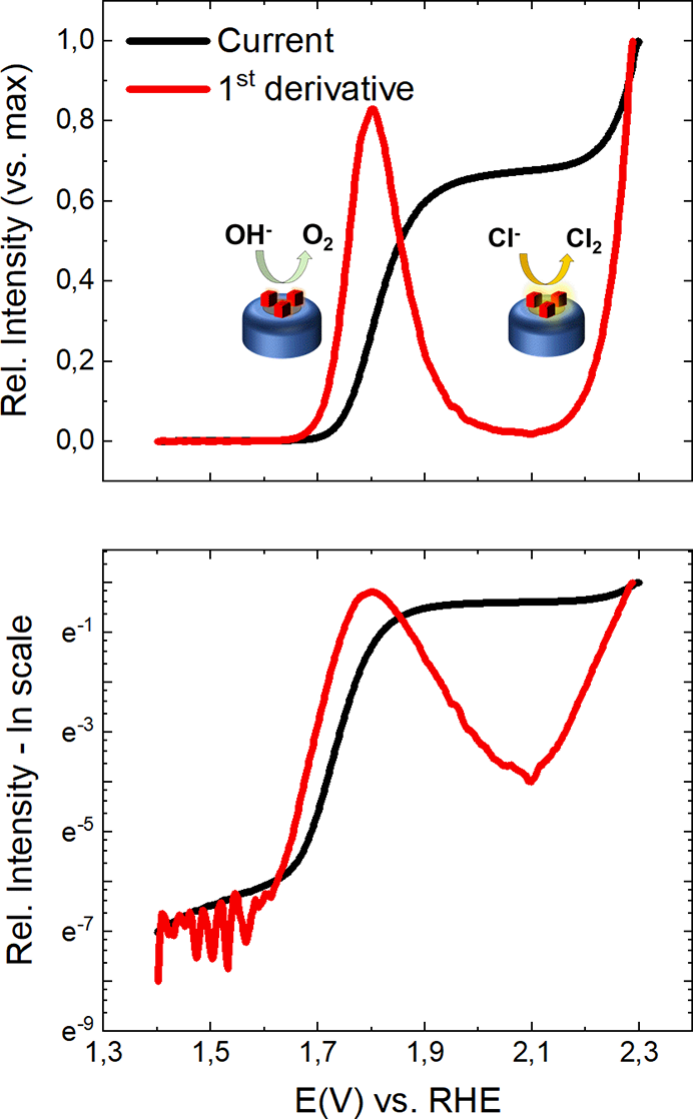
Normalized experimental LSV data (5 mV/s scan rate, 2 min equilibration at OCP) are collected from a 10 μm Pt UME in a 100 mM KCl + 1.0 mM KOH deaerated solution. The electrode has been functionalized with 36 nm Co_3_O_4_ nanoparticles by dip coating for 10 s in a 0.4 mg/mL solution. The normalized first derivative is reported in red. Corresponding semilogarithmic plots are reported in the lower panel. A second exponential growth is clearly observed above 2.1 V in the first derivative data (lower panel, red), while its presence is not obvious in the ln(*i*) – *E* plot (lower panel, black).

At last, we finally highlight the capability of this derivative-based approach to investigate a broad range of potentials at once. In Figure 4, we present a similar analysis performed on a 10 μm diameter Pt-UME dip-coated in a dispersion of cubic 36 nm Co_3_O_4_ nanoparticles (Co_3_O_4_/Pt UME) whose TEM characterization is reported in Section SI.7 and Figure S8. The OER activity of the system has been investigated in a 100 mM KCl + 1.0 mM KOH solution. While in Figure 4 only a single slope is easily recognized in the ln (*i*) – *E* plot, two linear regions are visible in the ln (*d*_1_) – *E* plot at around 1.7 and 2.1 V vs RHE, respectively. This agrees with an OER process initially proceeding through OH^–^ conversion at potentials smaller than 1.8 V, which is then limited by mass transport and reaches a steady state between 1.8 and 2.1 V. In the same potential range, an exponential onset of lower slope is observed on the pristine Pt UME in agreement with the moderate catalytic properties of Pt for the OER in alkaline solutions (see Figure S9A,B). However, above 2.1 V, the current loses it exponential character. Another exponential profile (that is, an electrochemical conversion limited by electron transfer) is observed instead for the Co_3_O_4_/Pt system (see Figure 4). The complexity of the system requires caution in attributing this second exponential growth to a specific reaction, as it could arise from either Co_3_O_4_ dissolution or oxidation, water splitting, or chlorine evolution. First of all, the exponential current increase, clearly visible thanks to the differential Tafel analysis developed herein, suggests a kinetically-controlled process. Then, cyclic voltammetry measurements and additional LSV experiments suggest that the system remains stable even upon reaching 2.3 V vs RHE (see Figure S9C,D). We would tentatively exclude that the second exponential profile at 1.8 V is related to oxidation/dissolution of either Pt or Co_3_O_4_ nanoparticles. No reduction current is observed, and each CV cycle overlaps with the previous ones. If during one CV the irreversible dissolution of the surface would occur, we would expect a detectable change in the subsequent cycles. This was however not observed (see Figure S9C,D). On the basis of these considerations, either direct water-splitting or chlorine evolution from Cl^–^ anions is suggested to occur at the Co_3_O_4_ nanoparticles.^31^ To distinguish between the hypothesized reactions, differential electrochemical mass spectrometry (DEMS) measurements were performed to determine the potential dependent reaction products (see Section SI.9 and Figure S10). As Cl_2_ was detected at potentials above 2.1 V (1.2 V vs Ag/AgCl-3 M KCl), the current increase at higher potentials can be assigned to chlorine evolution.

## Conclusions

In summary, we highlight how the extraction of charge-transfer coefficients by Tafel analyses requires a correction of the collected electrochemical current data and a proper choice of the considered potential ranges. Unfortunately, the quality of both these steps cannot be easily gauged as no benchmarking quantity is generally available to validate the analysis procedure. In this Perspective, we suggest such a benchmark by exploiting the derivatives of the current to obtain simple, reliable, and robust information about the performed kinetic analysis. In fact, not only must the derivatives share the exponential dependency (i.e., the same charge-transfer coefficient) with the original current data, but they also allow one to infer which mechanisms affect the electrochemical currents. This is proven for simulated single-electron transfers, experimental cases studies on HER and OER processes on Pt microelectrodes, and is then exploited to extract kinetic data from a Co_3_O_4_ nanocatalyst functionalized Pt support. Since derivation can be applied to almost any existing data, it emerges as a powerful tool to corroborate and compare Tafel slope measurements with recent and past literature. Automated data evaluation may benefit from this tool in the context of data mining and artificial intelligence methods currently emerging in electrocatalysis research. If supported by microkinetic considerations, the reliably obtained rate information may additionally significantly speed up the recognition of surface reaction mechanisms during electrochemical conversions. For these reasons, we suggest the differential approach as a possible path for both unambiguous recognition and faster development of high-performing electrocatalyst materials.

## Supporting Information Available

COMSOL numerical simulations of electrochemical processes, TEM and SAED data of Co_3_O_4_ nanoparticles, electrochemical data of OER on Co_3_O_4_ nanoparticles on Pt, and DEMS data on Co_3_O_4_ nanoparticles on Pt (PDF)
