# Supplementary material for: Differential Tafel Analysis: A Quick and Robust Tool to Inspect and Benchmark Charge Transfer in Electrocatalysis
Source: ACS Catal. 2022 Oct 27;12(21):13805–12. doi: 10.1021/acscatal.2c03581 (PMC9638995; doi:10.1021/acscatal.2c03581)
Supplement: Supplementary file 1 — cs2c03581_si_001.pdf [file cs2c03581_si_001.pdf]

Supporting Information

Viewpoint: Differential Tafel Analysis — a Quick and Robust Tool to Inspect and Benchmark Charge-transfer in Electrocatalysis

Manuel Corva[1], *Niclas Blanc*[1], *Christoph J. Bondue*[1], *Kristina Tschulik** [1,2]

*Corresponing author: kristina.tschulik@rub.de

Address

1 Analytical Chemistry II, Faculty of Chemistry and Biochemistry, Ruhr University Bochum, Bochum, 44780, Germany

2 Max-Planck-Institut für Eisenforschung GmbH, Max-Planck-Straße 1, Düsseldorf, 40237, Germany

Table Of Contents

SI.1: Potential conversion and mesh for numerical simulations 2
SI.2: Approximation of Butler Volmer equation to a single exponential term 3
SI.3: Potential-dependent mass transport and background corrections 5
SI.4: Goodness-of-fit parameters: a limited guarantee of proper charge transfer coefficient evaluation. 6
SI.5: Differential Tafel analysis and differential Tafel plots 7
SI.6: Applying derivative operator to currents presenting a kinetic contribution 9
SI.7: Co_3O_4 nanoparticle synthesis and characterization 10
SI.8: Cyclic voltammetry of Co_3O_4/Pt in 1.0 mM KOH + 100mM KCl 11
SI.9: DEMS response of the Co_3O_4/Pt system in OH^-/Cl^- containing solutions 12
References 13

SI.1: Potential conversion and mesh for numerical simulations

Potential conversion

The measured potentials in the case of 1.0 M KOH and 0.5 M H_2SO_4 solutions have been converted into potentials versus reversible hydrogen electrode (RHE). This was performed adding the open circuit potential (OCP) measured against a commercial RHE electrode stored for at least one day in the same solution. The measured OCP values were 0.70 V and 0.93 V for the 0.5 M H_2SO_4 and the 1.0 M KOH solution, respectively.

For the 1 mM KOH + 100 mM KCl solution, the potential conversion was performed according to

E(RHE) = E(Ag/AgCl) + 0.21 V + 0.059 V × pH = E(Ag/AgCl) + 0.86 V.

Numerical simulations

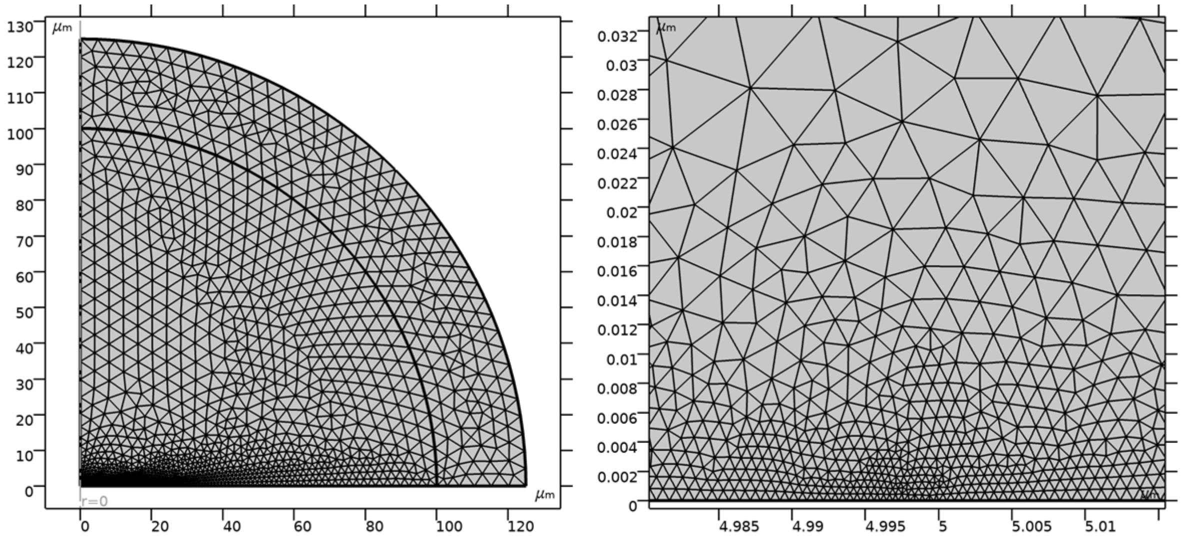

Figure S 1. The custom mesh, which was used for the simulation in 2D-space is shown. The disc-shaped microelectrode had a radius of 5 µm, and the inner domain a radius of 100 µm. Outwards, an infinite element domain with default scaling settings was used. The right image shows the edge of the electrode, which needed to be meshed extremely fine to avoid large influences of the singularity located at this point.

SI.2: Approximation of Butler Volmer equation to a single exponential term

In its complete form, the Butler Volmer (BV) equation consists of a cathodic and anodic term each characterized by an exponential profile. In some specific cases one of the two terms is negligible with respect to the other, as for example at high overpotentials or in the case of irreversible reactions.[1–3] However, it is possible to extract useful information about the charge transfer coefficient also in other conditions, as for example if the pre-exponential terms (i.e. the p_i parameters) are sufficiently small. To prove this, let us consider the formulation of the BV equation commonly considered in analytical electrochemistry

$$j_{\mathrm{K}} = \mathrm{p_a} e^{\alpha_{\mathrm{a}} f(E-E_{\mathrm{r}})} - \mathrm{p_c} e^{-\alpha_{\mathrm{c}} f(E-E_{\mathrm{r}})} = k_0 \left(c_{s,a} e^{\alpha_{\mathrm{a}} f(E-E_{\mathrm{f}})} - c_{s,c} e^{-\alpha_{\mathrm{c}} f(E-E_f)}\right), \quad (1)$$

where α_i are the anodic and cathodic transfer coefficients respectively, f is a placeholder for $\frac{F}{RT}$ with Faraday's constant F, the gas constant R and the absolute temperature T. The reference potential E_r is set to the formal potential E_f and the p_i terms can be expressed as the electrochemical rate constant $k_0$and the surface concentrations $c_{s,i}$ according to

$$p_i = k_0\, c_{s,i}. \quad (2)$$

From Eq.*(2)*, it is clear that p_i linearly depends on both k_0 and $c_{s,i}$. Thus, there exist possible combinations of k_0 and $c_{s,i}$ so that the pre-exponential parameter p_i are small enough to sufficiently "separate" the cathodic and anodic contributions in the current-potential plot.

To demonstrate how small values of p_i might allow to observe clear potential dependencies, we simulate the electrochemical response of a chemically reversible redox couple undergoing a one-electron conversion at a 10 µm ultramicroelectrode (UME). The formal potential has been set to 0 V, and the concentration of both the reduced and oxidized forms has been set to be equal. Two different rate constants (left and right panels) have been considered to highlight this "separation" effect, and the results are reported in Figure S 2.

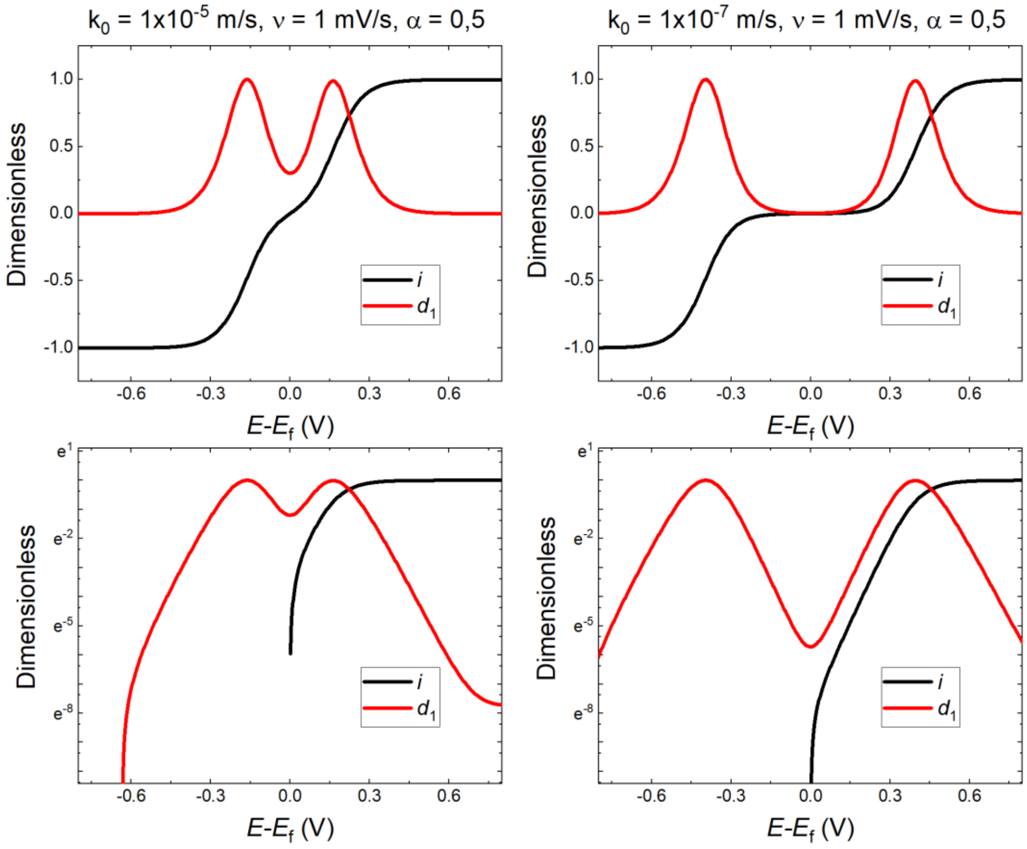

Figure S 2. Simulated LSV response of a one-electron redox couple over a 10 µm UME. Equimolar concentrations of both the reduced and oxidized form are considered. From left to right, the rate constant is decreased by two orders of magnitude. This induces an apparent separation of the exponential contributions, which can be easily recognized and inspected exploiting the derivative approach discussed in the main text; i = dimensionless current, d_1=first derivate of the current.

SI.3: Potential-dependent mass transport and background corrections

Constant-current backgrounds, originating for example from non-faradaic or steady-state processes, must be removed for the correct evaluation of charge-transfer coefficients from semi-logarithmic plots. At times, this task might be non-trivial. Two oxidation reactions occurring at two different formal potentials represent a useful example. This is showcased in Figure S 3, where the simulated current response of two distinct redox couples undergoing a one-electron oxidation on a 10 µm UME is reported. The two different formal potentials have been set to -0.6 and 0.1 V, respectively. Only the bulk concentration of the reduced form of each redox couple has been set different from zero. Such concentrations have been set to be identical for both reduced species.

If the semilogarithmic plot of the simulated current i (right panel, black line of Figure S 3) is inspected, a straight line emerges below -0.3 V, as expected when considering currents ruled by kinetics only. However, the second kinetic contribution around 0.3 V appears to be strongly distorted, if not inaccessible at all. This originates from the steady state oxidation current contribution due to the first redox couple, which introduces a constant background with respect to the second process. To access the kinetics of the redox couple of higher formal potential, a new iteration must be performed, and a different compensation must be implemented. The resulting dataset is reported in red in Figure S 3. Unfortunately, this means that both the kinetic terms cannot be accessed at the same time under the same correction. In this sense, the background correction evaluated at potentials below -0.3 V is hardly useful. Following the differential approach suggested in this work, it is possible to address the kinetics of both reactions at the same time, as showcased in Figure 4 of the main text.

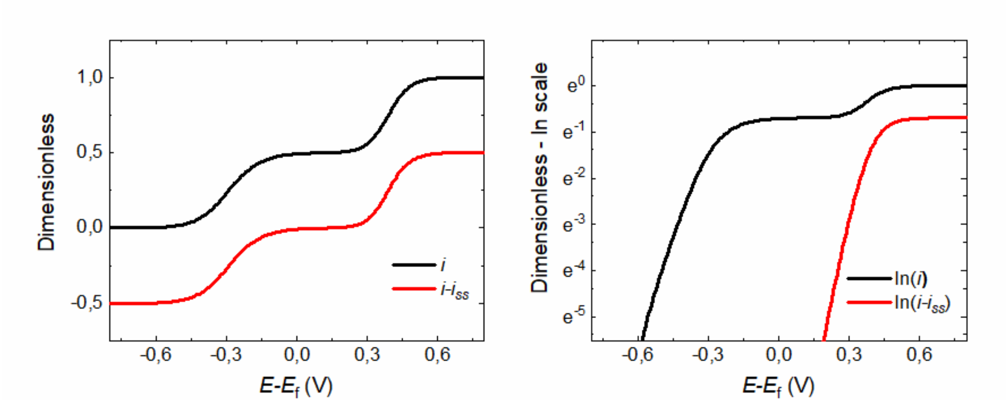

Figure S 3. Simulated LSV response of two reversible one-electron redox couples undergoing oxidation at a 10 µm UME. Equimolar concentrations of the reduced forms of both the redox couples have been considered, while the initial concentration of the oxidized species has been set to zero. Only after subtraction of the steady state current contribution (i_{ss}) of the first redox process the kinetics of the second can be accessed.

SI.4: Goodness-of-fit parameters: a limited guarantee of proper charge transfer coefficient evaluation.

To evaluate the kinetic parameters of a redox process, a practical approach consists in searching for a specific potential range over which the semi-logarithmic plot of the current upon the applied potential ($\ln(I)$ vs. E) shows a linear dependence. This approach is at the core of Tafel analysis. If this condition is found, then the charge transfer coefficient can be evaluated by linear fitting. Unfortunately, the logarithm of an exponential function is sensitive to any constant offset in the background, as shown in Figure 1 in the main text. We report in Figure S 4 the same dataset, but we focus our attention on the results of linear fitting performed on the background-corrected or uncorrected current values. As in the main text, a 1 % constant background has been added to the simulated current response of a micro electrode. The distortion introduced by the constant term leads not only to a restricted linear $\ln(I)$ vs. E region but also to an erroneous measurement of the kinetic parameter via linear fitting. An example of this is found in Figure S 4 C. Over the 0.05-0.15 V potential range, the estimation of the transfer coefficient value α_a appears to be straightforward in both the current datasets. Very small deviations from straight lines are observed, as confirmed by R^2 values and standard deviations associated to the estimated (anodic) charge transfer coefficient α_a, as reported in *Table 1* which contains the results of the linear fitting procedure. However, if the results are compared to the simulation parameter $\alpha_a^{th} = 0.6$, both estimations emerge as not correct. The current matches a straight line well, due to the complementary effect of both mass transport and background distortions, acting on the two opposite sides of the investigated potential range. The contribution from mass transport is hardly noticeable in Figure S 4 C and will be instead revealed and discussed in section SI.5 (see in Figure S 7 and Table 2) when derivatives will be considered.

To summarize: the goodness-of-fit parameter R^2 guarantees that a straight line is indeed well-matching the considered dataset, but it does not address the quality of the dataset itself, i.e. it cannot distinguish if the linearity is affected by external contributions.

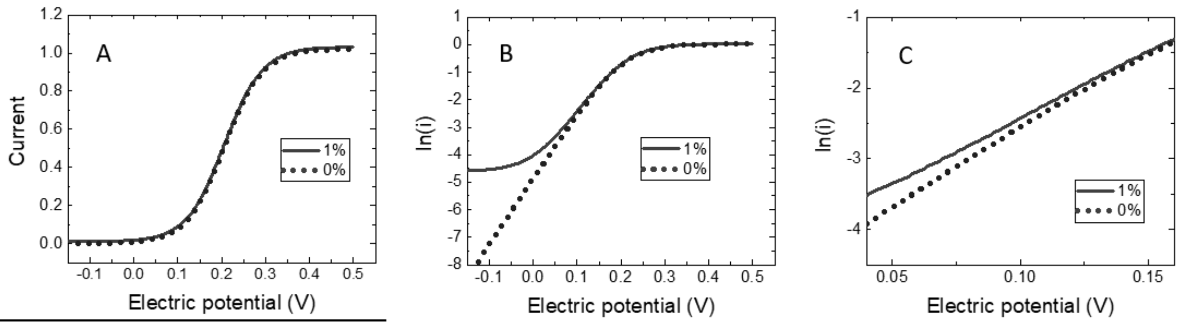

Figure S 4. Simulated microelectrode response without (dotted line) and with (solid line) the addition of a 1 % constant shift. In A), a linear current-potential plot is reported, while in B, C) the semi-logarithmic current-potential plot is reported over two different potential ranges to showcase the effect of the constant background. In particular in C), the distortion is such that a still linear region can be found although its slope is distorted and leads to false estimations of the charge transfer coefficient.

Table 1 Results from linear fitting of data in Figure S 4 *C.*

Constant term	$\alpha_a f$ (V^{-1})	S. DEV. (V^{-1})	R^2	α_a	S.DEV.
0 %	21.89	0.07	0.9995	0.561	0.002
1 %	19.02	0.04	0.9998	0.488	0.001

SI.5: Differential Tafel analysis and differential Tafel plots

Recently, Roth and coworkers[4] introduced the concept of differential Tafel plots to enhance the sensitivity and reliability of extraction of charge transfer coefficients from mass-transport and background corrected datasets. While exploiting a differential approach, the method allows to carefully check the quality of the extracted charge-transfer values overcoming the limits of classical linear fitting procedures. For this reason, it represents a significant and valuable asset to enhance the quality of the extracted kinetic parameters from high quality data.

However, the enhanced sensitivity of the approach makes it more dependent on the quality of the collected data. In Figure S 5 we showcase the results obtained applying the differential Tafel plot method reported in [4] and the differential Tafel analysis introduced in this work. The electrochemical current produced by a single electron irreversible reaction occurring at a micro electrode is analytically modelled by means of the following equations

$$I_{\mathrm{K}} = k_0\, \pi r^2 e^{\alpha_{\mathrm{a}} f(E)}, \tag{3}$$

$$I_{\mathrm{MT}} = 4\mathrm{FcDr}, \tag{4}$$

$$I_{\mathrm{M}} = \frac{I_{\mathrm{K}} I_{\mathrm{MT}}}{I_{\mathrm{K}} + I_{\mathrm{MT}}}, \tag{5}$$

where the parameters

k_0	Rate constant (1/s)	1E-4
r	Microelectrode radius (m)	2E-6
α_{a}	Charge transfer coefficient	0.4
f	F/RT	39.5
C	Bulk concentration (mol/m^3)	1
D	Diffusion coefficient (m^2/s)	5E-9

are used and a constant background and white noise reported in Figure S 5 B are then added. The presence of noise, background and mass-transport limitations is indicated by the labels "Noise", "BG", and "MT", respectively. To compare the effect of noise, the noise level has been doubled for one of the calculated profiles (Double Noise). The analytical expressions used to calculate the dataset presented in Figure S 5 are presented in the following table

Used random noise (-1 to +1)	rnd
Double Noise	$2 * rnd * 10^{-12} + \pi r^2 k_0 e^{\alpha_{\mathrm{a}} fE}$
Noise	$rnd * 10^{-12} + \pi r^2 k_0 e^{\alpha_{\mathrm{a}} fE}$
Noise – BG	$(5 + rnd) * 10^{-12} + \pi r^2 k_0 e^{\alpha_{\mathrm{a}} fE}$
Noise – BG – MT	$(5 + rnd) * 10^{-12} + (\pi r^2 k_0 e^{\alpha_{\mathrm{a}} fE} * 4FDCr)/(\pi r^2 k_0 e^{\alpha_{\mathrm{a}} fE} + 4FDCr)$

From Figure S 5 C and Figure S 5 D it is possible to immediately notice the presence of mass-transport limitations, affecting potentials higher than 0.7 V for the NBM case. If mass transport is not present (i.e. if Eq. (5) is not considered), the effect of a constant background is still evident in Figure S 5 C, while it is already countered in the corresponding data in Figure S 5 D. If also the constant background is absent, the effects of pure noise can be observed in both Figure S 5 C and D. However, while the constant background is automatically removed and does not require further adjustments for differential Tafel analysis, in the case of Tafel plots the user might mistake the deviations due to the noise level for a non-perfectly compensated background. This dependency on noise stems from the non-linear response of the logarithm, which is especially amplified towards the negative direction. Thus, a non-constant signal very close to zero induces very strong fluctuations in the logarithmic scale.

Such an effect can lead to even further disturbances, as demonstrated in Figure S 6, where the background has been correctly removed, but the presence of both noise and of mass-transport effects deeply distort the apparent charge-transfer parameter.

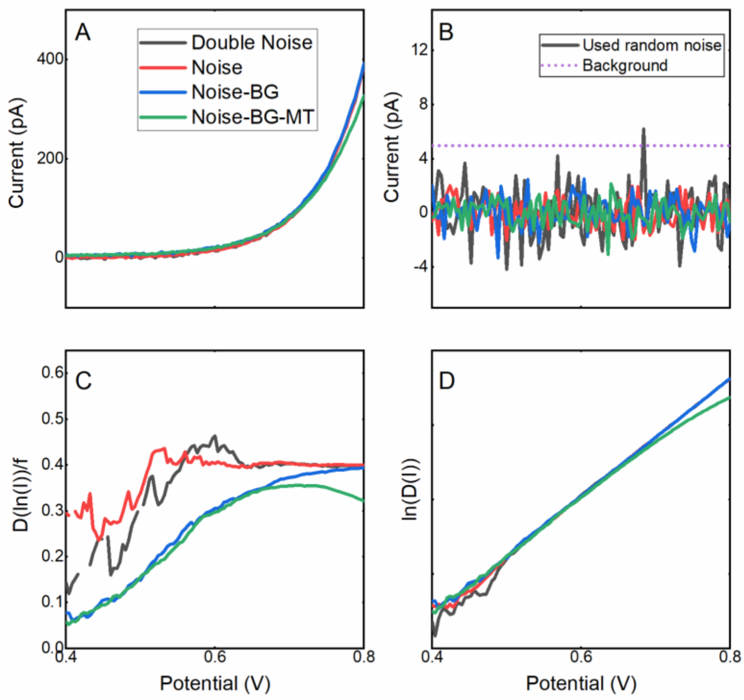

Figure S 5. Analytical kinetic current profiles expected from a single electron chemically irreversible reaction occurring on a microelectrode have been calculated with Eq.(3). To this, random noise of two different amplitudes (Double Noise and Noise), a constant background (Noise-BG, see panel B) and mass-transport limitations (Noise-BG-MT, according to Eq. (4) and (5)) have been added. Differential Tafel plot (panel C) and differential Tafel analysis (panel D) have been applied to the calculated dataset. Comparison of curves Double Noise, Noise and Noise-BG in panel C highlights the difficulty of differential Tafel plots to distinguish between noise in the dataset and uncompensated background effects, which instead are automatically removed by differential Tafel analysis in panel D.

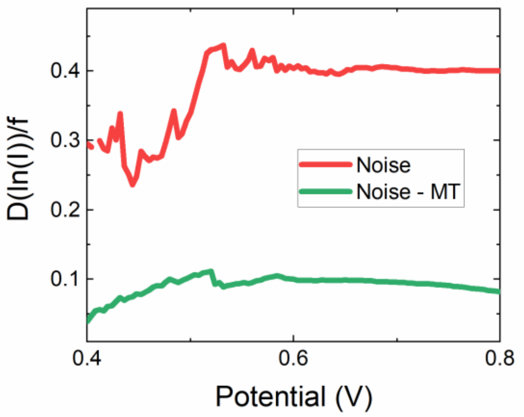

Figure S 6. Analytical kinetic current profiles expected from a single electron chemically irreversible reaction occurring on a microelectrode have been calculated by means of Eq.(3). To this, random noise (Noise) and mass-transport limitations (Noise-MT, according to Eq. (4) and (5)) have been added. The calculated dataset has been presented via differential Tafel plot. Comparison of curves Noise and Noise-MT highlights the sensitivity of differential Tafel plots to noise, particularly unfavourable when other uncompensated effects might be affecting the electrochemical currents at higher potentials (as mass-transport in this example).

SI.6: Applying derivative operator to currents presenting a kinetic contribution

It is possible to straightforwardly remove any constant contributions (as capacitive currents) by inspecting the derivative of the current versus the applied potential ($\frac{\partial I(E)}{\partial E}$). While any constant term will disappear upon derivation, the exponential contribution of any kinetic term is preserved due to the properties of the exponential function under derivation, according to

$$\frac{\partial I_{(E)}}{\partial E} = \frac{\partial(\text{const}+I_{\text{K}\,(E)})}{\partial E} = \frac{\partial(\text{const}+p_i \text{e}^{\alpha_\text{a} f(E-Er)})}{\partial E} = \alpha_\text{a} f \, I_{\text{K}(E)} \quad (6)$$

where the total current $I_{(E)}$ has been assumed to result only from the kinetic contribution ($I_{\text{K}(E)}$) of an anodic process, where α_a is the anodic charge-transfer coefficient, the potential of reference Er and the parameters p_i depend on the specific formulation of choice (see Dickinson et al.[3] for additional details) and $f = \frac{F}{RT}$ is used for brevity, where F and R refer to the Faraday and gas constant, respectively, and T to the temperature.[3]

The benefits of the differential approach can be seen in Figure S 7, where the same dataset is considered as in Figure S 4, but the semi-logarithmic plot of its derivative is reported. The presence of the background does not induce any appreciable effect and the two curves overlap. Moreover, the estimation of the α_a value becomes more reliable as the derivative highlights where the approximation of pure kinetic currents starts to fail. As mentioned in the main text, when mass transport starts to play a significant role, the derivative deviates from its exponential increase and correspondingly its semi-logarithmic plot deviates from a straight line. Even over the limited potential range considered before in Figure S 4 C and reported again in Figure S 7 C, a distortion from a linear trend can be distinguished and corresponds to a decreasing R^2 value (see Table 2 and compare with Table 1). As the results obtained over this potential range are not compatible, the obtained charge-transfer coefficient is probably inaccurate and the analysis should be improved. For example, a proper potential range unaffected by mass transport should be considered after correct background compensation.

To summarize: in the present case the decreased R^2 value suggests that there is no linear relationship. Considering that the derivative approach removed all constant background contributions, it is likely that mass transport corrections have to be implemented or a different potential range should be considered.

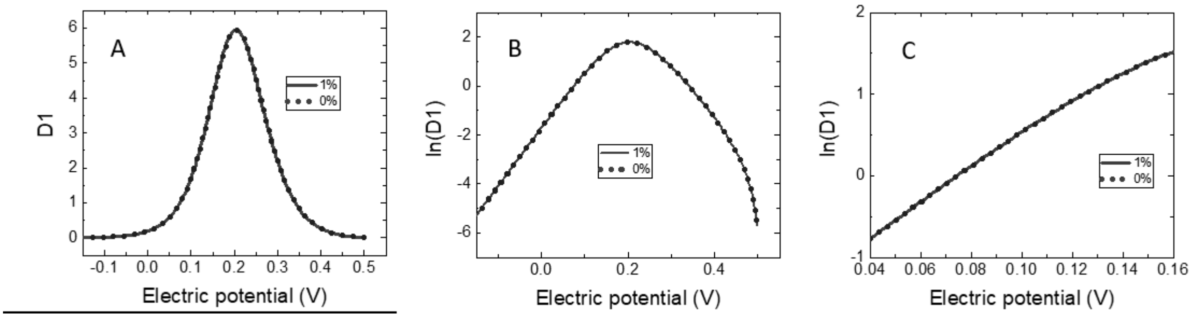

Figure S 7 Derivative of the dataset presented in Figure S 4. The ln-linear plot in C shows some deviations from linearity, suggesting that over this potential range we approach the failure of the purely kinetic model and have some influence from mass transport. The comparison between a 0 % and 1 % background cases is presented to highlight that no distortion is obtained through derivation.

Table 2 Results from linear fitting of data in Figure S 7 C.

Constant term	$\alpha_\text{a} f$ (V^{-1})	S. Dev. (V^{-1})	R^2	α_a	S. Dev.
0 %	19.8	0.2	0.997	0.508	0.005
1 %	19.8	0.2	0.997	0.508	0.005

SI.7: Co_3O_4 nanoparticle synthesis and characterization

Nanoparticle characterization

Transmission electron microscopy (TEM), high resolution TEM (HRTEM) and selected area electron diffraction (SAED) were carried out on JEOL JEM-2200FS operating at 200 kV. Samples for TEM studies were prepared by depositing an aqueous suspension of Co_3O_4 nanoparticles onto a carbon coated 200-mesh copper grid.

Nanoparticle synthesis

To synthesize the Co_3O_4 cubic nanoparticles, 0.1 g (0.4 mmol) cobalt acetate tetrahydrate were dissolved in 25 mL H_2O and transferred into 150 mL glass autoclave. After sealing, the solution was heated in a preheated 120 °C oil bath under continuous stirring for 1 h. Afterwards, it was centrifuged at 11000 rpm for 10 min. The supernatant was removed, and the nanoparticles were re-suspended in H_2O and centrifuged again. This was repeated three times more to remove the unreacted salt. At last, the nanoparticles were collected after drying overnight at 60 °C. TEM and SAED characterizations are reported in Figure S 8, together with the measured size distribution.

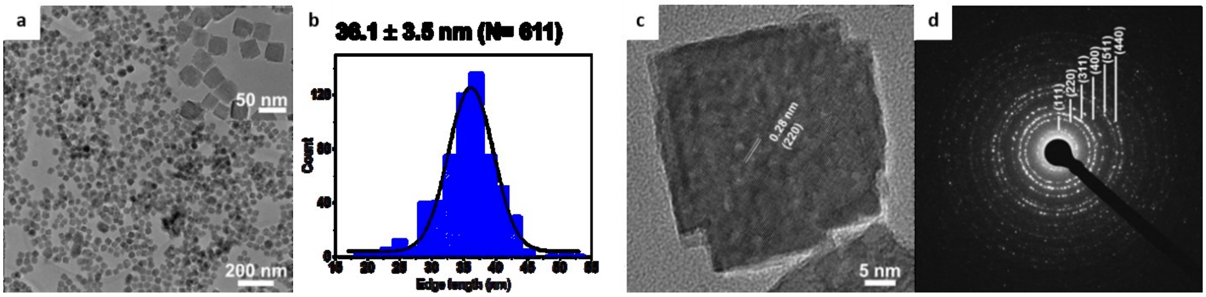

Figure S 8. a) TEM, b) edge length distribution, c) HRTEM and d) SAED pattern of non-capped Co_3O_4 36 nm cubes.

SI.8: Cyclic voltammetry of Co_3O_4/Pt in 1.0 mM KOH + 100mM KCl

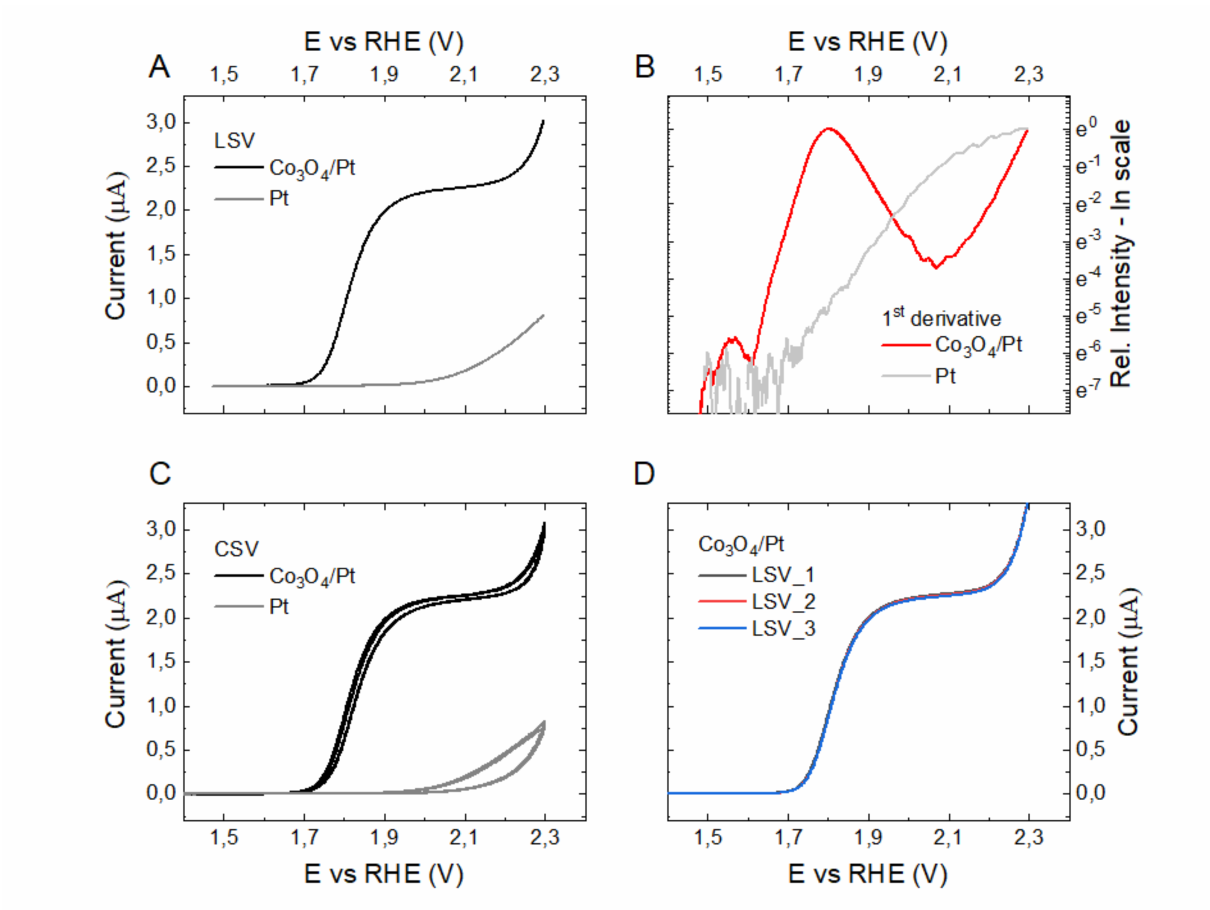

Figure S 9. Linear sweep voltammetry (LSV, 5 mV/s scanrate, 2 minutes equilibration at OCP) and cyclic voltammety (CV, 5 mV/s scanrate, 2 minutes equilibration at OCP, 3 cycles) data collected in 100 mM KCl + 1.0 mM KOH deareated solution on a 10 µm Pt UME. The electrode has been used either as polished or after functionalization with 36 nm Co_3O_4 nanoparticles by dip coating for 10 s in a 0.4 mg/mL solution. A) Comparison of Pt and Co_3O_4/Pt UME response. B) differential Tafel analysis of the LSV response from Pt and Co_3O_4/Pt UME. C) CVs of Pt and Co_3O_4/Pt UME. The overlapping current signal and the absence of reduction currents suggest no (significant) modifications of the Co_3O_4/Pt sistem even at high (2.3 V vs RHE) potentials. D) Additional LSV measurements on the Co_3O_4/Pt UME further support this picture. We thus exclude the current onset at around ca 2.1 V vs RHE to originate from corrosion or surface oxidation. Water splitting or chlorine evolution might explain this feature, while the steady state contribution establishing around 1.9 V is attributed to OH^- oxidation.

SI.9: DEMS response of the Co_3O_4/Pt system in OH^-/Cl^- containing solutions

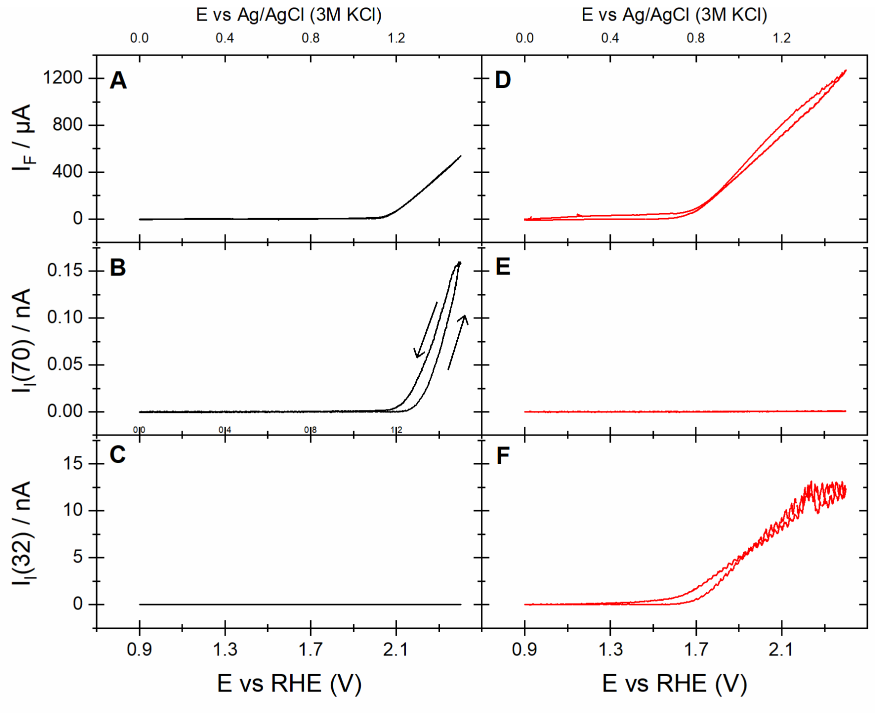

Figure S 10. DEMS measurement conducted on 36 nm Co_3O_4 nanoparticles drop cast on a macroscopic Pt-electrode. Panel A shows the CV obtained in 0.5 M KCl and Panels B and C show the mass spectrometric response for mass 70 (Cl_2) and 32 (O_2). The curves in panels D, E and F are the corresponding responses from a Co_3O_4/Pt electrode immersed in a 0.5 M KCl + 10 mM NaOH solution. At higher pH values, the mass 70 (Cl_2) signal is likely not detected due to the chlorine disproportionation reaction in alkaline environment.

Figure S 10 reports the electrochemical current and mass spectrometry response obtained from 36 nm Co_3O_4 nanoparticles drop cast on a thin film of Pt sputtered-deposited on a glass disc and immersed either in 0.5 M KCl or in 10 mM NaOH + 0.5 M KCl solutions. In the absence of NaOH, the CV features only one anodic process, which becomes noticeable above 2.1 V. The absence of a signal in the ionic current for mass 32 (Figure S 10 C) indicates that no water splitting occurs, whereas the strong signal in the ionic current for mass 70 indicates the oxidation of Cl^- to Cl_2 (Figure S 10 B).

The red curve of Figure S 10 D shows the first CV that was obtained at a freshly prepared electrode in an electrolyte containing both 500 mM KCl and 10 mM NaOH. As the potential passes a value of 1.5 V the current begins to increase monotonically. At low overpotentials, the ionic current for mass 32 largely follows this behavior, which suggests that oxygen evolution is the only reaction occurring in this potential range. However, as a potential of *ca.* 2.1 V is reached, the growth of the ionic current for mass 32 appears to slow down, if compared with the almost constant increase in the faradic current. Since oxygen evolution cannot then account for the constant increase in the faradic current, this suggests that additional electrochemical processes begin to take place. Considering the interpretation of Figure S 10 A, B and C, these additional electrochemical processes are most likely related to Cl_2 evolution again. In fact, the absence of a signal in the ionic current for mass 70 is likely attributed to Cl_2 disproportionation in alkaline media to Cl^- and ClO^-. These ions do not pass from the aqueous to the gas phase and are therefore not detected by via mass spectrometry. Hence, no signal in the ionic current for mass 70 (Figure S 10 E) despite Cl_2 evolution being expected.

Despite the realistic occurrence of Cl_2 evolution at potentials beyond 2.1 V, there is a potential window between 1.5 and 2.1 V in which the anodic current appears to be only due to oxygen evolution. Hence, any Tafel slopes derived in this potential should correspond to the oxygen evolution reaction only.

References

(1) Li, D.; Batchelor-McAuley, C.; Compton, R. G. Some thoughts about reporting the electrocatalytic performance of nanomaterials. *Applied Materials Today* **2020**, *18*, 100404. DOI: 10.1016/j.apmt.2019.05.011.
(2) Li, D.; Lin, C.; Batchelor-McAuley, C.; Chen, L.; Compton, R. G. Tafel analysis in practice. *Journal of Electroanalytical Chemistry* **2018**, *826*, 117–124. DOI: 10.1016/j.jelechem.2018.08.018.
(3) Dickinson, E. J.; Wain, A. J. The Butler-Volmer equation in electrochemical theory: Origins, value, and practical application. *Journal of Electroanalytical Chemistry* **2020**, *872*, 114145. DOI: 10.1016/j.jelechem.2020.114145.
(4) Khadke, P.; Tichter, T.; Boettcher, T.; Muench, F.; Ensinger, W.; Roth, C. A simple and effective method for the accurate extraction of kinetic parameters using differential Tafel plots. *Sci Rep* **2021**, *11* (1), 8974. DOI: 10.1038/s41598-021-87951-z. Published Online: Apr. 26, 2021.
